# Oridonin Suppresses Colorectal Cancer Growth In Vitro and In Vivo: Evidence from Integrated Transcriptomic and Proteomic Profiling

**DOI:** 10.3390/cimb48050440

**Published:** 2026-04-23

**Authors:** Menglong Xu, Yongchao Li, Wenqiang Sun, Haocheng Guan, Tinghui Wu, Shuwei Li

**Affiliations:** 1 Department of Biochemistry and Molecular Biology, College of Life Science and Technology, the State Key Laboratory Incubation Base for Conservation and Utilization of Bio-Resource in the Tarim Basin of the Xinjiang Production and Construction Corps, Tarim University, Alar 843300, China; xumenglong1989@163.com (M.X.);; 2 School of Medical Technology, Xinjiang Hetian College, Hetian 848000, China

**Keywords:** colorectal cancer, oridonin, transcriptomic, proteomic, apoptosis

## Abstract

Colorectal cancer (CRC) remains a major cause of cancer-related mortality worldwide, and effective therapeutic options for advanced disease are still limited. Oridonin (ORI), a naturally derived diterpenoid compound, has shown anti-tumor activity in several malignancies, but its molecular mechanisms in CRC remain incompletely understood. In this study, the anti-cancer effects of ORI were evaluated in HT-29 and HCT116 colorectal cancer cells using in vitro assays, integrated transcriptomic and proteomic analyses, Western blotting, and an HT-29 xenograft model. ORI reduced cell viability in a time- and concentration-dependent manner, induced G1-phase cell cycle arrest, increased cell death, and reduced wound closure under the tested in vitro conditions. Integrated omics analyses in HT-29 cells identified extensive alterations in gene and protein expression, with significant enrichment of pathways related to cell cycle regulation and apoptosis. Western blotting further showed that ORI increased the expression of BAX, BID, CYCS, and CASP3 while decreasing BCL2 expression. In vivo, ORI significantly inhibited tumor growth in nude mice bearing HT-29 xenografts. These findings indicate that ORI suppresses CRC growth through coordinated regulation of cell cycle progression and apoptosis and suggest that ORI may serve as a potential therapeutic candidate for colorectal cancer.

## 1. Introduction

Colorectal cancer (CRC) remains a major global health burden and is among the leading causes of cancer-related morbidity and mortality worldwide [1,2]. Recent epidemiological data indicate that CRC ranks third in incidence and second in mortality globally, with approximately 1.9 million new cases and 904,000 deaths each year [2]. Clinically, CRC is characterized by a high risk of invasion, metastasis, recurrence, and treatment failure, particularly in advanced-stage disease [3,4,5,6,7,8]. Although multimodal treatment strategies, including surgery, chemotherapy, radiotherapy, and targeted therapy, have improved patient management, durable therapeutic responses remain limited in many cases. Thus, the development of effective therapeutic agents with defined molecular mechanisms is still urgently needed.

Oridonin (ORI) is a naturally occurring diterpenoid isolated from *Rabdosia rubescens* and is widely used as a bioactive component in traditional Chinese medicine [9,10,11]. ORI has attracted increasing interest because of its anti-inflammatory, antioxidant, and anti-tumor properties. It has been reported to inhibit NLRP3 inflammasome activation and reduce the release of pro-inflammatory cytokines such as IL-1β and TNF-α [12,13]. Moreover, ORI exerts anti-cancer effects in several tumor types, including breast cancer, non-small-cell lung cancer, and thyroid cancer, through mechanisms involving oxidative stress regulation, apoptosis induction, and suppression of malignant progression [14,15,16,17,18].

In CRC, ORI has been shown to induce apoptosis, modulate autophagy, and alter cancer cell metabolism [19,20,21]. However, current evidence is still largely based on single-pathway studies, and a systematic understanding of the molecular networks regulated by ORI is lacking. In particular, no study has comprehensively integrated transcriptomic and proteomic data to define the anti-CRC mechanisms of ORI.

To address this gap, we investigated the effects of ORI on colorectal cancer cells and explored its underlying molecular basis using an integrated multi-omics strategy. The effects of ORI on cell viability, cell cycle progression, cell death, and wound closure were examined in HT-29 and HCT116 cells. Transcriptomic and proteomic profiling was then performed in ORI-treated HT-29 cells, and representative apoptosis-related proteins were validated by Western blotting. In addition, the anti-tumor activity of ORI was evaluated in an HT-29 xenograft model in nude mice. Our findings provide integrated evidence that ORI suppresses CRC growth through coordinated regulation of cell cycle- and apoptosis-related pathways.

## 2. Materials and Methods

### 2.1. Reagents and Cell Cultures

ORI (Cat. No. B21396) was obtained from Yuanye Biotechnology (Shanghai, China) and kept at 4 °C. The colorectal cancer cell lines HT-29 (Cat. No. CL-0118) and HCT116 (Cat. No. CL-0096) were provided by Procell (Wuhan, China). HT-29 cells were cultured in McCoy’s 5A medium, whereas HCT116 cells were cultured in Dulbecco’s Modified Eagle Medium (DMEM); both media contained 10% fetal bovine serum (FBS) and 1% penicillin–streptomycin (PS). Cultures were maintained at 37 °C with 5% CO_2_ in a humidified incubator. FBS (Cat. No. 164210), penicillin–streptomycin (Cat. No. PB180120), and DMEM (Cat. No. PM150210) were purchased from Procell (Wuhan, China). HT-29 and HCT116 cells were selected because they are well-established and widely used human colorectal cancer cell lines with distinct biological characteristics, allowing evaluation of the anti-tumor effects of ORI across different CRC cellular contexts. In addition, HT-29 cells were used for xenograft experiments because of their stable tumorigenic capacity in vivo.

### 2.2. Cell Proliferation Assay

Cell viability was assessed by a CCK-8 assay. HT-29 and HCT116 cells were plated in 96-well plates at a density of 3 × 10^4^ cells per well and allowed to adhere overnight. HT-29 cells were treated with ORI at final concentrations of 0, 6, 8, 10, 12, 14, 16, 18, and 20 μM, while HCT116 cells were treated with ORI at final concentrations of 0, 5, 10, 15, 20, 25, and 30 μM; both cell lines were treated for 24 or 48 h in their respective culture media. After treatment, the culture supernatant was removed, and 100 μL of fresh medium containing 10% CCK-8 reagent (Cat No. CA1210; Solarbio, Beijing, China) was added to each well for incubation at 37 °C for 1.5–2.0 h. Absorbance was then recorded at 450 nm using a K6600A+ microplate reader (Beijing Kao Technology Development Co., Ltd., Beijing, China).

Each treatment condition was performed in triplicate, and all experiments were repeated at least three times independently (*n* = 3). Cell viability (%) was calculated as [optical density (OD)-treated/OD-control] × 100%. IC_50_ values were determined in GraphPad Prism 9.5 by fitting the concentration–response data to a sigmoidal dose–response (variable slope) model [log(inhibitor) vs. normalized response, four-parameter logistic model (4PL)]. The corresponding IC_50_ values were 15.17 μM (95% CI: 14.90–15.44 μM) and 11.14 μM (95% CI: 10.82–11.46 μM) for HT-29 cells at 24 and 48 h, respectively, and 17.59 μM (95% CI: 16.72–18.46 μM) and 14.16 μM (95% CI: 13.39–14.94 μM) for HCT116 cells at 24 and 48 h, respectively. For most subsequent mechanistic experiments, the 24 h IC_50_ values were used because this exposure duration produced a clear biological response while preserving sufficient viable cells for the assessment of early cellular and molecular events, thereby reducing potential confounding effects associated with prolonged cytotoxic exposure.

### 2.3. Cell Cycle Analysis

HT-29 and HCT116 cells (2 × 10^5^ per well) were cultured in 6-well plates and treated with ORI using IC_50_-matched concentrations (15.17 μM for HT-29; 17.59 μM for HCT116). An equal volume of dimethyl sulfoxide (DMSO) was added to control wells. After 24 h exposure, cells were collected by trypsin digestion and fixed with 70% ethanol pre-cooled on ice, with fixation performed at −20 °C overnight. Cells were rinsed in PBS, resuspended, and stained for 20 min in the dark with ribonuclease A (RNase A) (2 μL, 1 mg/mL) together with propidium iodide (PI) (50 μL, 100 μg/mL). Samples were analyzed on a FACS Calibur flow cytometer (BD Biosciences, San Jose, CA, USA), and FlowJo_V10 (BD Life Sciences, Ashland, OR, USA) software was used to calculate the percentages of cells in G1, S, and G2 phases.

### 2.4. Cell Apoptosis and Necrosis Detection

HT-29 and HCT116 cells were plated at a density of 2 × 10^5^ cells/well in 6-well culture dishes and exposed to ORI at concentrations of 15.17 μM (HT-29) and 17.59 μM (HCT116) for 24 h. Control wells received an equivalent volume of DMSO as a vehicle. At the end of the treatment period, the culture supernatant was removed and cells were rinsed once with PBS. Apoptosis and necrosis were detected by staining with the YO-PRO-1/PI dual-fluorescence assay kit (Cat No. C1075S; Beyotime Biotechnology, Shanghai, China) at 37 °C under light-protected conditions for 20 min. Fluorescence images were subsequently captured using a Nikon A1RHD25 & N-SIM imaging system (Nikon, Tokyo, Japan), and fluorescence intensity was quantified using ImageJ software (version 1.53) (National Institutes of Health, Bethesda, MD, USA).

### 2.5. Cell Migration Assay

A scratch wound healing assay was performed to assess the effect of ORI on cell migration. HT-29 and HCT116 cells were grown to a confluent monolayer in 6-well plates. An artificial gap was introduced by drawing a 200 μL pipette tip steadily across the cell surface. After two to three PBS washes to remove floating cells, fresh serum-free medium supplemented with ORI at 15.17 μM (HT-29) and 17.59 μM (HCT116) was added. Phase-contrast images of the wound region were captured at 0 h and 48 h using a Nikon Eclipse Ci-L microscope (Nikon, Tokyo, Japan). For each well, images at 0 h and 48 h were obtained from the same wound region, and wound width was measured in ImageJ. The percentage of wound closure was calculated as (initial wound width — remaining wound width)/initial wound width × 100%.

### 2.6. Western Blot Analysis

Total proteins from HT-29 and HCT116 cells were isolated using Radioimmunoprecipitation Assay (RIPA) lysis buffer containing Phenylmethylsulfonyl Fluoride (PMSF) (RIPA:PMSF = 100:1), following the manufacturer’s protocol. Protein samples were fractionated by Sodium Dodecyl Sulfate-Polyacrylamide Gel Electrophoresis (SDS-PAGE) and subsequently blotted onto polyvinylidene fluoride (PVDF) membranes (Cat. No. IPVH00010; Millipore, Billerica, MA, USA). Membrane blocking was performed for 15 min at room temperature using a ready-to-use blocking reagent (Cat. No. PS108; Epizyme, Shanghai, China). The blots were then incubated at 4 °C overnight with the following primary antibodies: BAX (Cat. No. 50599-2-Ig, 1:5000), CYCS (Cat. No. 10993-1-AP, 1:2000), BID (Cat. No. 10988-1-AP, 1:2000), CASP3 (Cat. No. 19677-1-AP, 1:1000), β-actin (Cat. No. 81115-1-RR, 1:5000) (all from Proteintech, Wuhan, China), and BCL2 (Cat. No. bs-0032R, 1:2000; Bioss, Beijing, China).

After washing the membranes thoroughly with Tris-buffered saline with Tween 20 (TBST) three times, a horseradish peroxidase (HRP)-conjugated goat anti-rabbit Immunoglobulin G (IgG) secondary antibody (Cat. No. bs-0295G-HRP, 1:5000; Bioss, Beijing, China) was applied for 1 h at room temperature. The membranes were washed an additional three times with TBST, and target protein bands were visualized using enhanced chemiluminescence (ECL) detection reagent (Cat. No. SQ101; Epizyme); chemiluminescent images were acquired using a Tanon 5200 chemiluminescence imaging system (Tanon Science & Technology, Shanghai, China). Densitometric quantification of band signals was conducted using ImageJ software, with β-actin as the endogenous control for equal loading verification.

### 2.7. RNA Sequencing and Analysis

HT-29 cells, a human colorectal carcinoma cell line, were grown in McCoy’s 5A medium fortified with 10% FBS and 1% PS. Once cell density reached 70–80% of the culture surface, ORI was administered at the IC_50_ value while parallel control wells received an equal volume of DMSO. After a 24 h incubation period, the medium was discarded and the adherent cells were rinsed thoroughly with PBS three times. Cells were harvested by gentle scraping, collected into sterile tubes, and sedimented via centrifugation at 500× *g* for 5 min under refrigerated conditions (4 °C). Each cell pellet was resuspended and lysed in 1 mL of TRIzol reagent (ensuring ≤ 10^7^ cells per sample) by repeated aspiration with a pipette until a transparent homogenate was obtained, and RNA extraction was performed according to the manufacturer’s instructions. RNA concentration was measured using a Qubit 4.0 Fluorometer (Thermo Fisher Scientific, Waltham, MA, USA), and RNA integrity was evaluated using a Qsep 400 Bio-Fragment Analyzer (BiOptic Inc., La Canada Flintridge, CA, USA); all samples exhibited RNA Integrity Number (RIN) values of 9.8.

Poly(A) mRNA was enriched using oligo(dT) magnetic beads and fragmented into short sequences. Double-stranded cDNA was synthesized, end-repaired, A-tailed, ligated with sequencing adapters, and amplified by PCR to construct the final cDNA library. Libraries were sequenced on an Illumina NovaSeq 6000 (Illumina, San Diego, CA, USA) platform with a paired-end 150 bp (PE150) strategy. A total of 77.29 Gb clean data was generated for 8 samples (*n* = 4 per group), with no less than 8 Gb clean data per sample and Q30 ≥ 96%.

Raw sequencing data were filtered using fastp (v0.23.2) to remove adapters and low-quality reads. Clean reads were mapped to the human reference genome GRCh38 (Ensembl 109) using HISAT2 (v2.2.1). Gene expression levels were quantified with featureCounts (v2.0.3). Differentially expressed genes (DEGs) were identified using DESeq2 (v1.38.3) with thresholds of adjusted *p*-value < 0.05 and |log_2_FC| ≥ 1 [22]. Gene ontology (GO) and KEGG pathway enrichment analyses were performed using the clusterProfiler (v4.6.0) package on the Metware Cloud analytical platform (https://cloud.metware.cn) (accessed on 10 February 2026).

### 2.8. Proteomic Sequencing and Analysis

HT-29 cells were cultured in McCoy’s 5A medium with 10% FBS and 1% PS and treated for 24 h with ORI at its 24 h IC_50_; parallel controls received the vehicle (DMSO). After treatment, cells were washed with ice-cold PBS and collected (500× *g*, 5 min, 4 °C) and pellets were frozen at −80 °C and shipped on dry ice to Metware Biotechnology (Wuhan, China) for global quantitative proteomics (*n* = 4 biological replicates per group).

Protein extraction and digestion: Cell pellets were lysed in 8 M urea, 50 mM ammonium bicarbonate, and 1 mM PMSF on ice and sonicated. Lysates were cleared (15,000× *g*, 10 min, 4 °C) and protein concentration was measured by bicinchoninic acid (BCA). For each sample, 100 μg protein was reduced with 5 mM dithiothreitol (DTT) (37 °C, 45 min), alkylated with 15 mM iodoacetamide (room temperature, dark, 30 min), diluted to ≤ 2 M urea, and digested with sequencing-grade trypsin (1:50, w/w) overnight at 37 °C. Peptides were desalted on C18 cartridges, dried, and reconstituted in 0.1% formic acid.

Liquid chromatography–tandem mass spectrometry (LC–MS/MS): Peptides were separated using Vanquish Neo ultra-high-performance liquid chromatography (UHPLC) (Thermo Fisher Scientific, Waltham, MA, USA) with an analytical C18 column (75 μm × 25 cm, 2 μm, 100 Å), using a 60-min gradient at 300 nL/min (mobile phase A: 0.1% formic acid in water; B: 0.1% formic acid in acetonitrile; column temperature 40 °C). The LC was coupled to an Orbitrap Astral mass spectrometer (Thermo Fisher Scientific, Waltham, MA, USA) operated in data-independent acquisition (DIA) mode. Key settings included: spray voltage 2.0–2.1 kV; ion transfer tube 320 °C; full MS resolution 120,000 (m/z 200); scan range m/z 350–1250; high-energy collisional dissociation (HCD) fragmentation; MS/MS resolution 30,000 with variable isolation windows covering the full mass range.

Data analysis: Raw files were processed in DIA-NN (v1.8.1) in library-free mode against the UniProt human reference proteome (UP000005640, 2023_05). The false discovery rate (FDR) was controlled at 1% at both precursor and protein levels, and protein groups required at least two unique peptides. Protein intensities were taken from DIA-NN reports with built-in cross-run normalization. DEPs were defined by *p* < 0.05 and |log_2_ fold-change| > 0.585 (i.e., fold-change ≥ 1.5 or ≤0.67). GO/KEGG enrichment was performed using the Metware Cloud Platform (https://cloud.metware.cn) (accessed on 10 February 2026).

### 2.9. Animal Experiments

Male BALB/c nude mice (6 weeks old, 18–20 g at the time of acquisition) were purchased from Sipeifu Biotechnology Co., Ltd. (Beijing, China). Animals were housed under Specific Pathogen-Free (SPF) conditions at 25 ± 0.5 °C with a relative humidity of 55 ± 2% under a 12 h light/12 h dark cycle, with free access to standard chow and water. After an acclimatization period of 7 days, a subcutaneous HT-29 xenograft model was established by inoculating 5 × 10^6^ viable cells into the right axillary subcutis of each mouse. When tumors reached approximately 50 mm^3^, mice were randomly allocated into two groups (*n* = 5 per group) using a random number table: a vehicle group (PBS containing 5% DMSO) and an ORI treatment group (20 mg/kg). The dose of ORI [20 mg/kg, i.p. (Intraperitoneal)] was selected based on a previous report using an HT-29 xenograft model [21]. ORI was administered intraperitoneally once every 3 days for 30 days, and control mice received the vehicle on the same schedule. Tumor length and width were measured with calipers at each dosing time point, and tumor volume was calculated as (length × width^2^)/2. Mice were monitored regularly throughout the study for general condition and tumor burden. The experiment was terminated if the tumor volume reached 1500 mm^3^. At the study endpoint, mice were euthanized by overdose of pentobarbital sodium, and tumors were excised for measurement and imaging. The experimental plan was written before study initiation but was not formally registered. All animal procedures were conducted with approval from the Science and Technology Ethics Committee of Tarim University (project license PB20250418003, approved on 18 April 2025) and followed institutional standards for animal welfare.

### 2.10. Statistical Analysis

Statistical analyses were performed using GraphPad Prism 9.5 (GraphPad Software, San Diego, CA, USA). Data are presented as mean ± SD. Differences between the two groups were analyzed using an unpaired two-tailed Student’s *t*-test. Normality was assessed using the Shapiro–Wilk test before applying the t-test. For in vivo tumor growth curves measured over time, two-way ANOVA was used. A *p* value < 0.05 was considered statistically significant. The specific statistical test used for each experiment is indicated in the corresponding Section 3 and figure legends. The statistical methods for in vitro and in vivo experiments are described separately above.

## 3. Results

### 3.1. ORI Suppresses the Viability of Colorectal Cancer HT-29 and HCT116 Cells In Vitro

**Figure 1:**
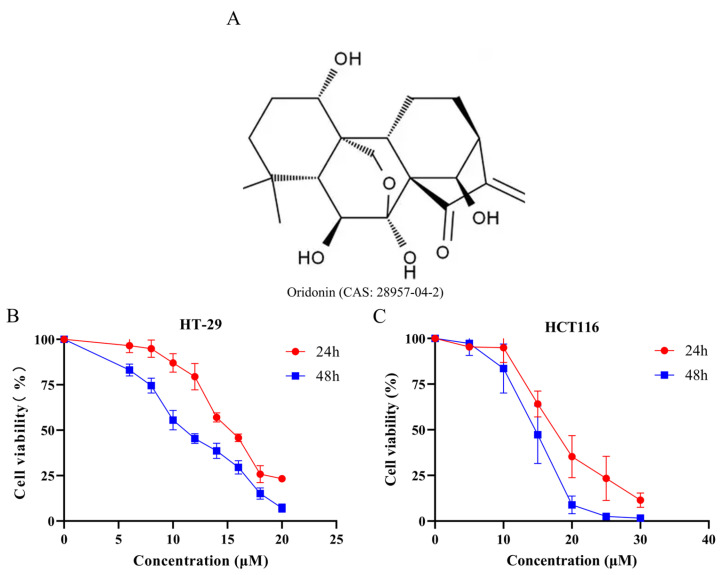
Evaluation of ORI-mediated growth inhibition in colorectal cancer cells. (**A**) Structural formula of ORI (CAS: 28957-04-2). (**B**,**C**) The growth-inhibitory activity of ORI against HT-29 (**B**) and HCT116 (**C**) cells was determined by CCK-8 assay at two time points (24 and 48 h). HT-29 cells were treated with ORI at concentrations of 0, 6, 8, 10, 12, 14, 16, 18, and 20 μM for 24 or 48 h, while HCT116 cells were treated with ORI at concentrations of 0, 5, 10, 15, 20, 25, and 30 μM. Red circles and blue squares represent cell viability at 24 and 48 h, respectively. All data are presented as mean ± SD (*n* = 3). Statistical significance was determined using an unpaired two-tailed Student’s *t*-test. CCK-8, Cell Counting Kit-8; ORI, oridonin; SD, standard deviation.

The growth-inhibitory effect of ORI on colorectal cancer cells was evaluated using the Cell Counting Kit-8 (CCK-8) assay. As shown in Figure 1B,C, treatment with increasing concentrations of ORI for 24 h and 48 h progressively reduced cell viability in both HT-29 and HCT116 cells, demonstrating clear time- and concentration-dependent effects. These results indicate that ORI effectively reduces the viability of colorectal cancer cells in vitro.

### 3.2. ORI Induces G1-Phase Cell Cycle Arrest in HT-29 and HCT116 Cells

**Figure 2:**
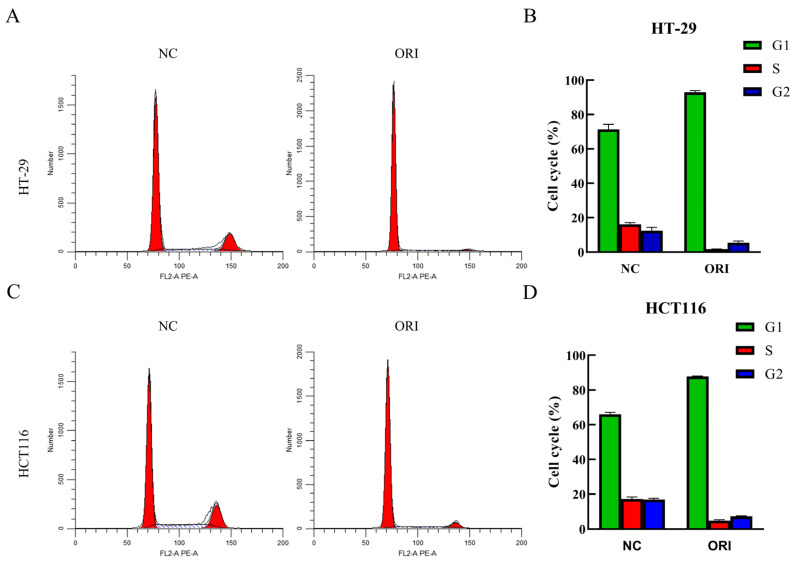
ORI induces cell cycle arrest in HT-29 and HCT116 cells. Cells were exposed to ORI (15.17 μM for HT-29 and 17.59 μM for HCT116, corresponding to their respective 24 h IC_50_ values) or an equivalent volume of DMSO vehicle for 24 h, and cell cycle status was evaluated using flow cytometry. (**A**,**C**) Flow cytometric histograms representative of cell cycle phase allocation in HT-29 and HCT116 cells under each treatment condition. (**B**,**D**) Bar graph summaries of the relative percentages of cells residing in G1, S, and G2 phases for HT-29 and HCT116 cells. All data are presented as mean ± SD (*n* = 3). Statistical significance is indicated as follows. DMSO, dimethyl sulfoxide; NC, negative control; ORI, oridonin; SD, standard deviation.

Flow cytometric profiling demonstrated a clear shift in cell-cycle distribution following ORI treatment (Figure 2A). In HT-29 cells, the G1 phase fraction was markedly elevated from 71.37% to 92.88% upon ORI exposure (*p* < 0.001), while the proportions of cells in the S phase (16.15% to 1.68%) and G2 phase (12.48% to 5.44%) were substantially reduced (Figure 2B). Likewise, ORI-treated HCT116 cells displayed a significant expansion of the G1 population (65.92% to 87.81%, *p* < 0.001), coupled with a decline in S phase (17.21% to 4.87%) and G2 phase (16.87% to 7.32%) cell fractions (Figure 2C,D). Under the tested treatment condition, ORI was associated with an accumulation of cells in the G1 phase, suggesting a potential effect on cell-cycle progression.

### 3.3. ORI Promotes Cell Death in Colorectal Cancer Cells, as Indicated by YO-PRO-1/PI Staining

**Figure 3:**
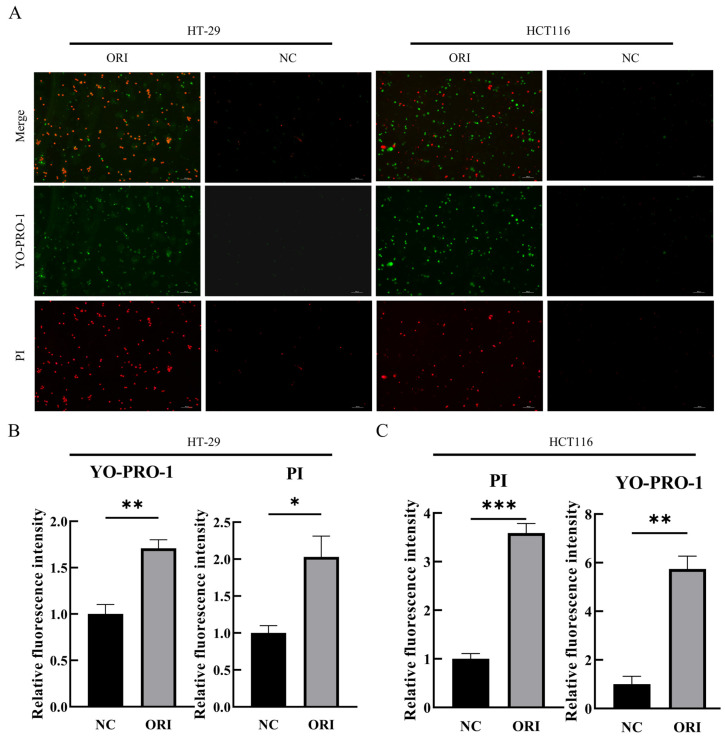
ORI increases YO-PRO-1 and PI fluorescence signals in HT-29 and HCT116 cells. HT-29 and HCT116 cells were treated with ORI (15.17 μM for HT-29; 17.59 μM for HCT116) or NC (DMSO vehicle) for 24 h and co-stained with YO-PRO-1 and PI. (**A**) Representative fluorescence images showing YO-PRO-1 (green), PI (red), and merged channels. Scale bar = 100 μm. (**B**,**C**) Quantification of the relative fluorescence intensities of YO-PRO-1 and PI in HT-29 (**B**) and HCT116 (**C**) cells, respectively. Data are presented as mean ± SD (*n* = 3). Statistical significance was determined using an unpaired two-tailed Student’s *t*-test. * *p* < 0.05, ** *p* < 0.01, *** *p* < 0.001.

The ability of ORI to induce cell death was examined in HT-29 and HCT116 cells using YO-PRO-1/PI double staining followed by fluorescence microscopy. Compared with the vehicle-treated group, ORI-treated cells showed a marked increase in green fluorescence intensity, indicating enhanced YO-PRO-1 uptake and suggesting the occurrence of early apoptotic changes (Figure 3). At the same time, red fluorescence intensity was also increased in the ORI-treated group, reflecting increased PI staining and indicating progression to late apoptosis and/or necrotic cell death. Because YO-PRO-1/PI staining alone cannot definitively distinguish specific cell death pathways, these findings were interpreted as evidence of increased cell death under ORI treatment. Together, these observations indicate that ORI increases cell death in colorectal cancer cells.

### 3.4. Effect of ORI on Wound Closure in HT-29 and HCT116 Cells

**Figure 4:**
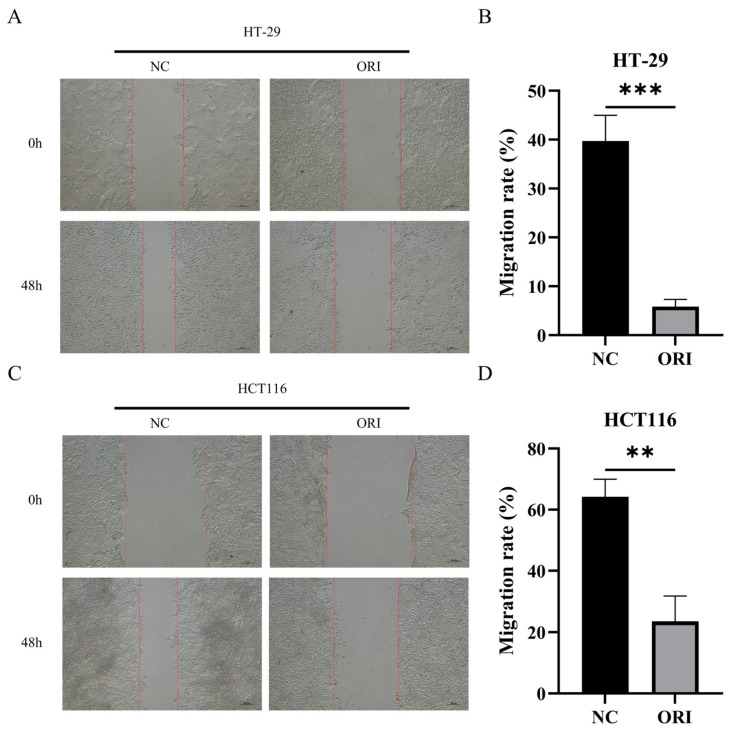
Effect of ORI on wound closure in HT-29 and HCT116 cells under the tested conditions. Images at different time points were captured from the same wound region. Cells were treated with ORI (15.17 μM for HT-29; 17.59 μM for HCT116) or NC (DMSO vehicle) in serum-free medium for 48 h. (**A**) Representative images of the wound region in HT-29 cells at 0 and 48 h. (**B**) Quantification of wound closure rates in HT-29 cells. (**C**) Representative images of the wound region in HCT116 cells at 0 and 48 h. (**D**) Quantification of wound closure rates in HCT116 cells. Scale bar = 100 μm. All data are presented as mean ± SD (*n* = 3). Statistical significance was determined using an unpaired two-tailed Student’s *t*-test. ** *p* < 0.01; *** *p* < 0.001. Because the assay was performed using IC_50_ concentrations in serum-free medium, reduced wound closure may partially reflect decreased cell viability and/or proliferation.

The effect of ORI on wound closure was assessed using a scratch wound healing assay. As shown in Figure 4A,B, ORI-treated HT-29 cells exhibited a markedly lower wound closure rate than control cells after 48 h, decreasing from 39.73% to 5.84% (*p* < 0.001). A similar effect was observed in HCT116 cells, in which the wound closure rate decreased from 64.24% in the control group to 23.52% in the ORI-treated group (*p* < 0.01) (Figure 4C,D). These results show that ORI markedly reduced wound closure in both HT-29 and HCT116 cells in the scratch assay.

### 3.5. Integrated Transcriptomic and Proteomic Analyses Reveal Extensive Molecular Changes Induced by ORI in HT-29 Cells

**Figure 5:**
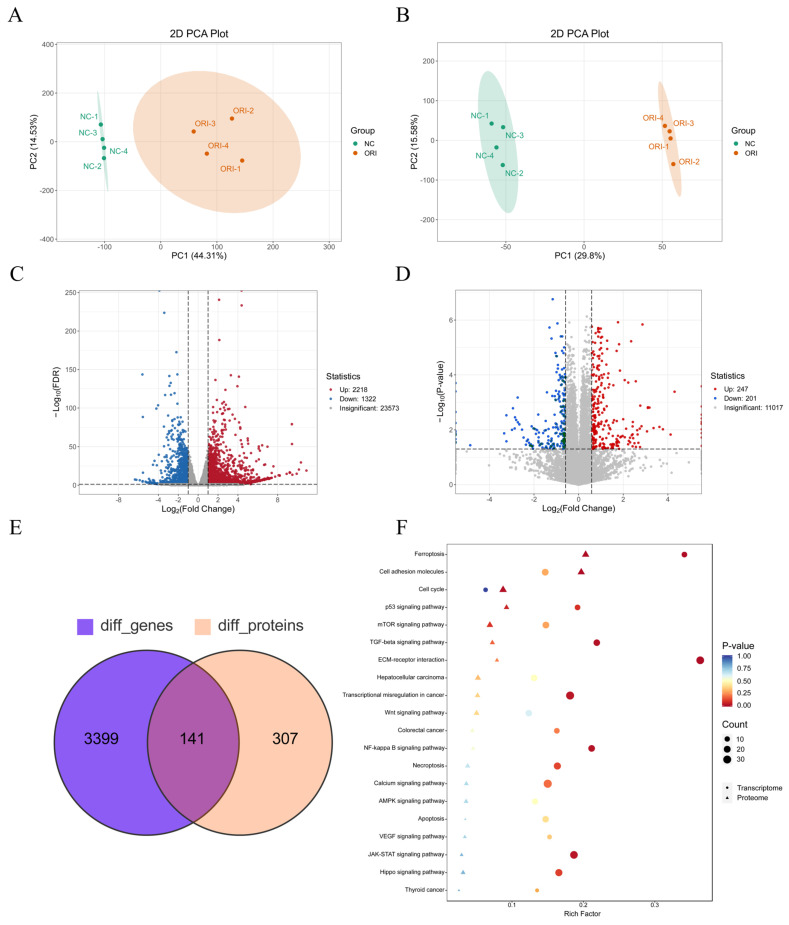
ORI-induced transcriptomic and proteomic changes in HT-29 cells. HT-29 cells were treated with ORI at 15.17 μM (IC_50_ at 24 h) or an equivalent volume of DMSO vehicle for 24 h (*n* = 4 biological replicates per group). (**A**) PCA plot showing transcriptomic profiles in HT-29 cells between the NC and ORI groups. (**B**) PCA plot showing proteomic profiles in HT-29 cells between the NC and ORI groups. (**C**) Volcano plot showing DEGs in HT-29 cells between the NC and ORI groups. (Padj < 0.05). Red indicates upregulated genes (*n* = 2218) and blue indicates downregulated genes (*n* = 1322). (**D**) Volcano plot showing DEPs in HT-29 cells between the NC and ORI groups (*p* < 0.05, |log2FC| > 0.585, corresponding to fold change (FC) ≥ 1.5). Dashed vertical lines indicate the log2FC cutoff of ±0.585. Red indicates upregulated proteins (*n* = 247) and blue indicates downregulated proteins (*n* = 201). (**E**) Venn diagram showing the overlap between DEGs and DEPs, identifying 141 molecules significantly altered at both the mRNA and protein levels. These overlapping molecules were used for downstream integrative pathway enrichment analysis. (**F**) KEGG pathway enrichment analysis was performed on the 141 overlapping molecules identified from the integrated transcriptomic and proteomic analysis (**E**). Both up- and down-regulated molecules were combined in the enrichment input. Dot/triangle size represents the number of mapped genes/proteins, and color indicates the enrichment *p*-value. DEGs, differentially expressed genes; DEPs, differentially expressed proteins; Diff, difference; ORI, oridonin; Padj, Adjusted *p*-value.

To further investigate the molecular mechanisms underlying the anti-tumor effects of ORI, integrated transcriptomic and proteomic analyses were performed in HT-29 cells. As shown in Figure 5A,B, principal component analysis (PCA) demonstrated clear separation between the NC and ORI groups in both transcriptomic and proteomic datasets, indicating that ORI induced substantial changes in gene and protein expression profiles. Transcriptomic analysis identified 2218 upregulated genes and 1322 downregulated genes in ORI-treated cells relative to the NC group (Figure 5C). Proteomic analysis identified 247 upregulated proteins and 201 downregulated proteins (Figure 5D). Integration of the two datasets revealed 141 overlapping molecules that were significantly altered at both the mRNA and protein levels (Figure 5E). Kyoto Encyclopedia of Genes and Genomes (KEGG) pathway enrichment analysis of these overlapping molecules showed significant enrichment in several cancer-related pathways, including “Cell cycle”, “Colorectal cancer”, and “Apoptosis” (Figure 5F). These findings indicate that ORI induces broad transcriptomic and proteomic alterations in HT-29 cells.

### 3.6. ORI Regulates Representative Apoptosis-Related Proteins in Colorectal Cancer Cells

**Figure 6:**
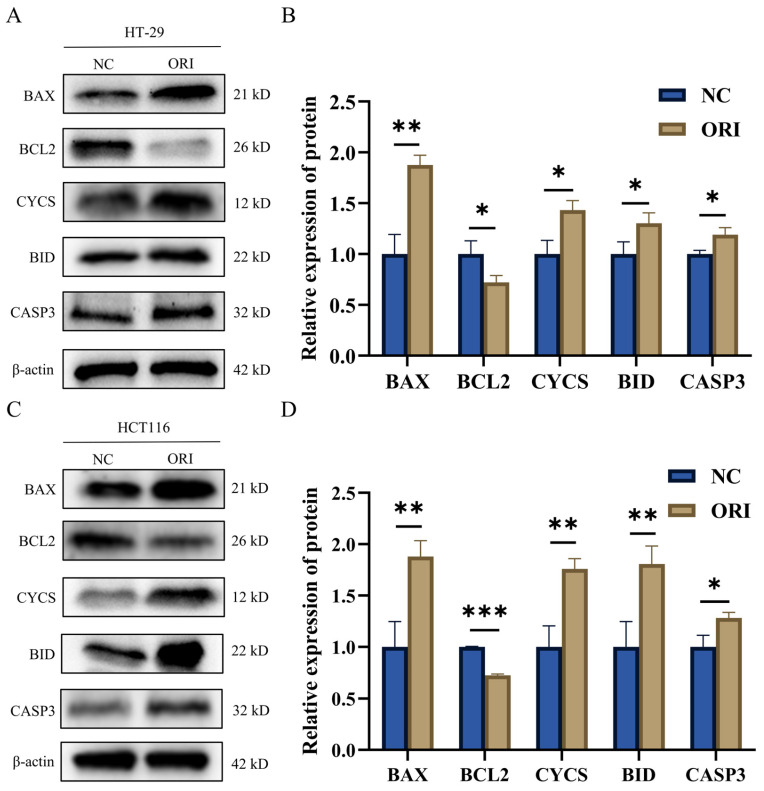
Western blot analysis of representative apoptosis-related proteins in CRC cells following ORI treatment. Protein selection was guided by the apoptosis pathway enriched among the 141 overlapping molecules identified in the integrated transcriptomic–proteomic analysis. HT-29 and HCT116 cells were treated with negative control (NC) or ORI (15.17 μM for HT-29; 17.59 μM for HCT116) for 48 h. (**A**,**B**) Representative blots (**A**) and quantitative analysis (**B**) of BAX, BCL2, CYCS, BID, and CASP3 expression in HT-29 cells. (**C**,**D**) Representative blots (**C**) and quantitative analysis (**D**) of the same proteins in HCT116 cells. β-actin was included as an internal standard. All data are presented as mean ± SD (*n* = 3). Statistical significance was determined using an unpaired two-tailed Student’s *t*-test. * *p* < 0.05; ** *p* < 0.01; *** *p* < 0.001. CRC, colorectal cancer; NC, negative control; ORI, oridonin; SD, standard deviation.

To validate the apoptosis-related signaling suggested by the integrated omics analysis, representative proteins enriched in the apoptosis pathway were examined by Western blotting in HT-29 and HCT116 cells after ORI treatment. As shown in Figure 6A–D, ORI increased the protein expression levels of BCL2-Associated X protein (BAX), BH3 Interacting Domain Death Agonist (BID), Cytochrome c (CYCS), and Cysteinyl Aspartate Specific Proteinase 3 (CASP3) while reducing the expression of B-Cell Lymphoma 2 (BCL2) in both cell lines. These results indicate that ORI regulates the expression of apoptosis-related proteins in colorectal cancer cells at the protein level.

### 3.7. ORI Inhibits Tumor Growth in an HT-29 Xenograft Model In Vivo

**Figure 7:**
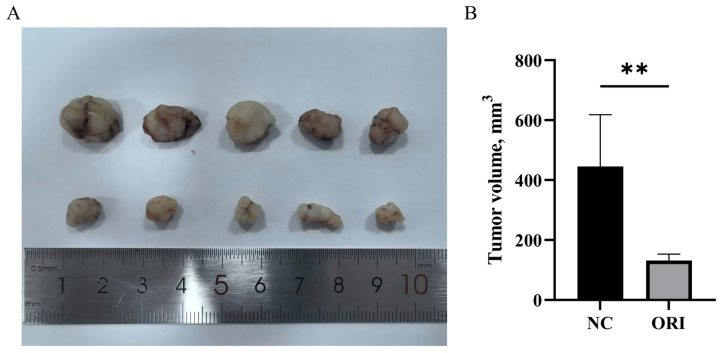
In vivo anti-tumor effect of ORI in nude mice. Mice bearing xenograft tumors were assigned to a vehicle group (NC: PBS + 5% DMSO) or an ORI group (20 mg/kg; intraperitoneal administration). (**A**) Representative images of tumors collected at the endpoint (vehicle, top; ORI, bottom). (**B**) Tumor volumes were measured and summarized for each group. Results are expressed as mean ± SD (*n* = 5). Statistical significance was determined using an unpaired two-tailed Student’s *t*-test. ORI treatment produced a significant reduction in tumor volume compared with NC (** *p* < 0.01). DMSO, dimethyl sulfoxide; NC, negative control; ORI, oridonin; PBS, phosphate-buffered saline; SD, standard deviation.

The anti-tumor activity of ORI in vivo was evaluated using a nude mouse xenograft model established with HT-29 cells. As shown in Figure 7A, tumors collected from the ORI-treated group were visibly smaller than those from the control group. Consistent with this observation, tumor volume measurements showed that ORI treatment significantly suppressed xenograft tumor growth compared with the control treatment (*p* < 0.01; Figure 7B). These findings indicate that ORI inhibits tumor growth in vivo in the HT-29 xenograft model.

## 4. Discussion

ORI has shown anti-tumor activity in multiple cancer types, but its effects in CRC have not been fully characterized. In the present study, ORI reduced the viability of HT-29 and HCT116 cells in a time- and concentration-dependent manner, induced G1-phase arrest, increased cell death, and reduced wound closure in vitro. In addition, ORI significantly inhibited tumor growth in an HT-29 xenograft model. At the molecular level, integrated transcriptomic and proteomic analyses identified broad changes in ORI-treated HT-29 cells, with enrichment of cell cycle and apoptosis pathways. These results were supported by Western blot analysis showing increased BAX, BID, CYCS, and CASP3 expression and decreased BCL2 expression. Together, these findings indicate that ORI exerts anti-CRC effects through coordinated modulation of cell cycle progression and apoptosis-related signaling.

Our results are in agreement with previous reports showing that ORI induces apoptosis in CRC cells. Yang et al. [19] reported that ORI triggered caspase-dependent apoptosis in HCT116 and LoVo cells through regulation of the Bim/BAX/BCL2 axis and the caspase-9/caspase-3 pathway. Consistent with these findings, we observed increased BAX and CASP3 expression and decreased BCL2 expression in both HT-29 and HCT116 cells. Compared with earlier pathway-focused studies, the present work provides a broader systems-level perspective by integrating transcriptomic and proteomic data. The overlap between these datasets and their convergence on cell cycle and apoptosis pathways strengthen the mechanistic interpretation of our results.

Previous studies have also suggested that ORI affects several cancer-related processes beyond apoptosis. ORI has been reported to promote ferroptosis in breast cancer cells through JNK/Nrf2/HO-1 signaling [14], induce apoptosis in non-small-cell lung cancer [17], and suppress epithelial–mesenchymal transition and angiogenesis in thyroid cancer [18]. In CRC, ORI has also been linked to autophagy and metabolic reprogramming [20,21]. Taken together, these studies suggest that ORI may act through multiple interconnected mechanisms. In our study, cell cycle- and apoptosis-related pathways emerged as the most prominent responses in the integrated omics analysis.

This study has several strengths, including the combined use of in vitro assays, in vivo validation, and integrated transcriptomic and proteomic profiling. To our knowledge, this is the first study to apply this multi-omics strategy to investigate ORI in CRC cells. In addition, the use of both HT-29 and HCT116 cells allowed the anti-tumor effects of ORI to be evaluated in two distinct CRC models.

Some limitations should be considered. Only two CRC cell lines were examined, and omics analysis was conducted only in HT-29 cells. Although major apoptosis-related changes were validated in both cell lines, future studies should include additional CRC models to better capture tumor heterogeneity and identify cell-line-specific responses. The xenograft model also has a limited ability to recapitulate the human tumor microenvironment, and in vivo toxicity was not systematically evaluated. Furthermore, because several assays were performed using IC_50_-based treatment conditions, some observed effects may have been influenced by cytotoxicity. In particular, the wound-healing results should be interpreted cautiously, as reduced wound closure may also reflect reduced viability and proliferation under the assay conditions.

Overall, our findings support ORI as a promising anti-CRC compound and provide integrated evidence that its effects are associated with disruption of cell cycle progression and apoptosis-related pathways. Further studies are needed to identify its direct molecular targets and evaluate its safety and translational potential.

## 5. Conclusions

In conclusion, the present study indicates that ORI exerts anti-tumor effects on CRC via diverse mechanisms, encompassing the inhibition of cellular proliferation, blockade of cell cycle progression at the G1 phase, facilitation of apoptotic and necrotic cell death, and reduced wound closure under the tested in vitro conditions. Collectively, these results highlight ORI as a promising candidate for further preclinical investigation in CRC. Additional studies are needed to define its safety profile, therapeutic window, and translational potential.

## Data Availability

The original contributions presented in this study are included in the article. Further inquiries can be directed to the corresponding author(s).

## Abbreviations

The following abbreviations are used in this manuscript:

Abbreviation	Full Name
ORI	Oridonin
CRC	Colorectal Cancer
CCK-8	Cell Counting Kit-8
IC_50_	Half Maximal Inhibitory Concentration
CI	Confidence Interval
DMSO	Dimethyl Sulfoxide
DMEM	Dulbecco’s Modified Eagle Medium
FBS	Fetal Bovine Serum
PS	Penicillin-Streptomycin
PI	Propidium Iodide
PBS	Phosphate-Buffered Saline
BAX	BCL2-Associated X protein
CYCS	Cytochrome c
BID	BH3 Interacting Domain Death Agonist
CASP3	Cysteinyl Aspartate Specific Proteinase 3
BCL2	B-Cell Lymphoma 2
DEGs	Differentially Expressed Genes
DEPs	Differentially Expressed Proteins
NLRP3	NOD-Like Receptor Pyrin Domain-Containing 3
IL-1β	Interleukin-1β
TNF-α	Tumor Necrosis Factor-α
JNK	c-Jun N-Terminal Kinase
Nrf2	Nuclear Factor Erythroid 2-Related Factor 2
HO-1	Heme Oxygenase-1
ROS	Reactive Oxygen Species
NSCLC	Non-Small-Cell Lung Cancer
EMT	Epithelial-Mesenchymal Transition
JAK2/STAT3	Janus Kinase 2/Signal Transducer and Activator of Transcription 3
PVDF	Polyvinylidene Fluoride
TBST	Tris-Buffered Saline with Tween 20
HRP	Horseradish Peroxidase
ECL	Enhanced Chemiluminescence
BCA	Bicinchoninic Acid
GO	Gene Ontology
KEGG	Kyoto Encyclopedia of Genes and Genomes
ANOVA	Analysis of Variance
SD	Standard Deviation
PCA	Principal Component Analysis
FC	Fold Change
FDR	False Discovery Rate
RIN	RNA Integrity Number
SDS-PAGE	Sodium Dodecyl Sulfate-Polyacrylamide Gel Electrophoresis
RIPA	Radioimmunoprecipitation Assay
PMSF	Phenylmethylsulfonyl Fluoride
SPF	Specific Pathogen-Free
4PL	Four-Parameter Logistic
i.p.	Intraperitoneal
NC	Negative Control
AMPK	AMP-Activated Protein Kinase
GLUT1	Glucose Transporter 1
MCT1	Monocarboxylate Transporter 1
PKM2	Pyruvate Kinase M2
AKT	Protein Kinase B
Padj	Adjusted *p*-value
DESeq2	Differential Expression Sequence 2
IgG	Immunoglobulin G
LC–MS/MS	Liquid Chromatography–Tandem Mass Spectrometry
DTT	Dithiothreitol
DIA	Data-Independent Acquisition
HCD	High-Energy Collisional Dissociation
UHPLC	Ultra-High-Performance Liquid Chromatography
RSL3	RAS-Selective Lethal 3
RNase A	Ribonuclease A
OD	Optical Density
